# The nuclear envelope in higher plant mitosis and meiosis

**DOI:** 10.1080/19491034.2019.1587277

**Published:** 2019-03-17

**Authors:** Monica Pradillo, David Evans, Katja Graumann

**Affiliations:** a Departamento de Genética, Fisiología y Microbiología, Facultad de Ciencias Biológicas, Universidad Complutense de Madrid, Madrid, Spain; b Department of Biological and Medical Sciences, Faculty of Health and Life Sciences, Oxford Brookes University, Oxford, UK

**Keywords:** Nucleus, mitosis, meiosis, higher plant, cell division, cell cycle, nuclear envelope, nuclear pores, phragmoplast

## Abstract

Mitosis and meiosis in higher plants involve significant reconfiguration of the nuclear envelope and the proteins that interact with it. The dynamic series of events involves a range of interactions, movement, breakdown, and reformation of this complex system. Recently, progress has been made in identifying and characterizing the protein and membrane interactome that performs these complex tasks, including constituents of the nuclear envelope, the cytoskeleton, nucleoskeleton, and chromatin. This review will present the current understanding of these interactions and advances in knowledge of the processes for the breakdown and reformation of the nuclear envelope during cell divisions in plants.

## Introduction

Higher plants undergo an open cell division in which the nuclear envelope (NE) breaks down to allow the mitotic spindle to connect with the chromosomes, exposing the genetic material to the cytoplasm. In common with animal cells, this means that completion of a successful cell division requires the breakdown and reformation of the nuclear envelope. Timing and positioning are crucial – and the proteins of nucleoplasm and cytoplasm which interact with the envelope are central to its completion. This review will focus on the current state of knowledge of these proteins, which are significantly different from those of animals, and their interactions during the processes of mitosis and meiosis, where relevant comparing them to those of other higher organisms.

The linker of nucleoskeleton and cytoskeleton (LINC) complex is central to protein interaction at the NE and greatest progress has been made in understanding this structure (reviewed by [1]. The LINC complex comprises SAD/UNC homology (SUN) domain proteins in the inner NE that bind with Klarsich Anc Syne Homology (KASH) domain proteins in the outer NE. Associated with these are proteins that connect with the nucleoskeleton and chromatin through interaction with the SUN domain proteins and with the cytoskeleton through connection with KASH domain proteins. The γ-tubulin complex (γ-TUC) also plays an important role in connecting the NE to the cytoskeleton [2], while the nuclear pore complexes (NPCs) interact with the nucleoskeleton as well as being anchored in the NE [3].

## The plant LINC complex

The higher plant LINC complex has recently been extensively reviewed [1,4] and therefore a brief summary will be presented here. Arabidopsis thaliana contains two SUN domain proteins in which the domain is C-terminal (C-SUNs) allowing SUN domain:KASH domain interaction, and three mid-SUNs (in most species [5]; in which the SUN domain is at a central position). All appear to be functional within the plant LINC complex, though mid-SUNs are also ER localized [1]. There is some evidence from expression patterns that some members of the SUN domain family, the mid SUNs AtSUN53 and ZmSUN3, might have specialized functions in meiosis [6,7] in addition to the C-ter SUNs which have a role in telomere anchorage [8].

In common with other multicellular organisms, while SUN domain proteins are structurally similar, much greater diversity is shown in the KASH domain proteins of the Outer NM (ONM); in animal systems, this provides for a diversity of interaction with different elements of the cytoskeleton [9] linked to the range of functions of the system. Identification of plant KASH proteins began with WIPs (for WPP domain interacting proteins) that were first described anchoring the Ran GTPase activating protein (RanGAP) to the NE [10], a function performed in non-plant systems by SUMOylation anchorage to the NPC [1]. WIPs (and the other plant KASHs) have a classical KASH structure in that they have a cytoplasmic coiled-coil domain, a transmembrane domain, and a short SUN-domain interacting sequence, in this case, the three amino acids VPT [1,10,11].

The entire complex required to anchor RanGAP has been shown to involve SUN and two WIPs (WIP1 and WIP2), two further proteins, the WPP domain-interacting tail-anchored proteins, WIT1 and WIT2 and may include further WPP proteins [10,12]. The next major group of plant KASH domain proteins described is termed SINEs (for SUN-interacting NE proteins). All five members of the family have VPT as the C-terminal sequence in common with the WIPs [13]. SINE 1 and 2 have cytoplasmic domains resembling armadillo repeats, while SINEs 3–5 have short, unique cytoplasmic domains [5]. Finally, an additional KASH domain protein, Toll Interleukin Receptor domain KASH protein, AtTIK, which appears to be unique to *Arabidopsis*, has been described during studies on interaction with the mid-SUN proteins [6,14]. AtTIK has a KASH domain more similar to non-plant systems with a characteristic PPPS motif as the final four amino acids [14]. A detailed phylogenetic analysis [5] suggests that all currently identified plant KASH proteins fall into the three groupings: SINEs, WIPs, and TIK and that a minimal plant LINC complex would comprise two mid-SUNs, one C-ter SUN, one SINE, and one WIP [5,13]. Recently, Gumber et al. [15], have provided a detailed description of LINC components in Zea mays L. including two additional KASH genes, *MLKG1* and *MLKG2* specific to grasses.

## The higher plant nuclear lamina

One key area of difference between animal and higher plant nuclei is in the structure of the nuclear lamina, classically described in animal cells as a layer of filamentous material made up of type V intermediate filaments, known as lamins [16]. These are multifunctional and bind to SUN domain proteins [17]. They play a key role in the onset of NE breakdown (NEBD) in cell division [18–20] where the release of the lamina from the envelope is a key event. Lamin homologues are not represented in the plant genome, though electron microscopy reveals a meshwork of fibers, initially called plamina, underlying and connected to the NE and interconnecting with the NPCs [3,21]. It is now widely accepted that the CRWN (for CRoWded Nucleus) family proteins [22] are major constituents of this meshwork and the main candidates to assume the functions of the lamins in plants since they are essential for maintaining nuclear size and shape [23–25]. They were first described as Nuclear Matrix Constituent Proteins, NMCP, in pioneering work in Masuda’s laboratory in carrots and Allium [26,27], and later in Arabidopsis they were first termed LINC for Little Nuclei [25], and then, to avoid confusion with the LINC complex described in the previous section, renamed CRWN because of the high nuclear DNA density displayed by the corresponding mutants [1,22]. Members of the family were present in a study by Sakamoto and Tagaki [28], in which mass spectrometry was used to identify more than 600 putative lamina components.

CRWN*s* have a long central coiled coil domain; in *Arabidopsis*. CRWN1 and CRWN4 were mainly localized to the nuclear periphery, whereas CRWN2 was in the nucleoplasm and CRWN3 was detected in both regions [25,28]. Interaction of CRWNs with the C-ter SUNs, SUN1 and SUN2 has been demonstrated [29]. In common with animal lamins, where mutants show small, deformed nuclei, *crwn1* and *crwn4* mutants have small and spherical nuclei [22,28] a property not seen for *crwn2* or *crwn3*. Plants with a *crwn1* mutation combined with *crwn2, crwn3,* or *crwn4* have even smaller nuclei [22].

## Other NE components involved in cell division

Recently other proteins have been identified which may provide additional linkages between the NE and chromatin in higher plants. The Nuclear Envelope Associated Protein (NEAP) family is conserved in angiosperms and gymnosperms [5] and has four members in Arabidopsis thaliana, one of which (AtNEAP4) appears to be a pseudogene [14]. NEAP structure consists of extensive coiled-coil domains, followed by a nuclear localization signal and a transmembrane domain predicted near the C-terminus. NEAPs interact with themselves and with both C-ter and mid-SUNs, while overexpression causes relocation of CRWN1 from the nuclear periphery to the nucleoplasm. NEAP interaction with a basic leucine zipper transcription factor AtbZIP18 has been observed [14].

A further putative component of the plant lamina is KAKU4, discovered in a mutant screen, where the mutants have smaller and more spherical nuclei. Overexpression results in NE overgrowth; the effect is more extreme when KAKU4 and CRWN1 are co-expressed [30]. Its role in the lamina has yet to be fully explored. Analysis suggests that the minimal functional angiosperm nucleoskeleton comprises one CRWN and one NEAP; KAKU4 is absent from some monocots and the primitive basal angiosperm *Amborella trichopoda* [5].

## The higher plant cytoskeleton: evidence for NE linkage

Interactions of the NE with the cytoskeleton are also of great importance during cell division, where the position of the nucleus as well as the breakdown and reformation of the NE are required in addition to the anchorage of chromatin and nucleoplasmic components. The KASH domain proteins described above interact with elements of the cytoskeleton. A mutant screen [31] revealed KAKU1, mysosin XI-i, one of the plant-specific myosin XI family, is associated with the NE. *kaku1* mutants have spherical nuclei. This protein binds to WIT1 and WIT2 and is believed to connect the plant LINC complex components WIP1/2/3 to the actin cytoskeleton [12]. As mentioned above, SINE1 directly interacts with actin via its ARM domain. These nucleus–actin interactions are essential in anchoring and moving the nucleus in various developmental processes [32,33].

Perhaps best described of the ONM components are the proteins of the γ-Tubulin complex. These take the place of centrosomes, which are absent in plants, provide anchorage points for microtubules (MTs) and are recruited to the NE as components of the mitotic spindle [34]. The complex comprises core subunits which bind to α-helical GCP-associated proteins. Mutation of GCP3-interacting protein 1 (GIP1) and GIP2, which bind to the core γ-tubulin complex protein 3 (GCP3), results in enlarged deformed nuclei and increased ploidy with redistribution of AtSUN1 in the INE and failure to form a functional mitotic spindle [2,35].

## The plant cell division cycle differs from that in other organisms with an open mitosis

The plant cell division cycle shows many features in common with other organisms; however, the presence of a cell wall and occurrence of endoreduplication (in which genome duplication occurs without division) means plant mechanisms are required to ensure the correct localization of the plane of division as well as its timing and conclusion. The presence of the cell wall prevents the mitotic swelling common in animal cells and in place of an actin-myosin driven constriction of the plasma membrane in cytokinesis, plant cells grow new plasma membranes and cell wall in a centripetal fashion to join the existing cell wall at a closely regulated location [36,37].

Higher plants also show interesting nuances in the use of the Ran system in cell division. Ran is a ubiquitous signal for the presence of chromatin (see [38] for a review) with high levels of RanGTP in the nucleoplasm and of RanGDP in the cytoplasm. The Ran guanine nucleotide exchange factor, RanGEF RCC1 remains active in mitosis and the RanGTP generated and associated with chromatin provides a spatial signal for the location of the mitotic spindle and reassembly of the NE [38]. RanGTP activating protein 1 (RanGAP1) is associated with the nucleoporin NUP358 (Ran-binding protein 2) through a SUMOylation mechanism throughout mitosis [39]. In higher plants, no equivalent of the SUMOylation/Nup binding mechanism appears to exist as RanGAP binding to the NE has been shown to involve SUN and KASH proteins (WIPs) as well as WPP domain-interacting tail-anchored proteins, WITs (see [40] and above). Plant RanGAP is involved in localizing the plane of division [41] and is a component of the membrane structure which forms the new dividing cell wall [42]. Thus, a role for components of the LINC complex in associating this key marker of chromatin in plants may be suggested.

Many of the other protein constituents of the NE involved in linking the NE and nucleoskeleton are not present in plants [1,4,43]. The lamin B receptor which is an inner NE membrane-intrinsic protein that binds to B-type lamins is absent as are proteins of the LEM domain family (LAP2, Emerin, Man1) that bind to chromatin through association with Barrier to Autointegration Factor (BAF). Breakage of these associations is by a phosphorylation mechanism; that between chromatin and LEM is the result of the action of protein kinase VRK1 (see [44]). It is likely, however, that the same underlying mechanism of phosphorylation to initiate membrane breakdown occurs in plants, and further research is needed to identify the protein interactors involved.

## From initiation of division to nuclear envelope breakdown

Overall control of division, in common with other organisms, lies in the control of the G1-S-G2-M cycle. Key regulators are conserved with cyclin-dependent kinases (CDKs) having a primary control of cycle progression [45]. Plant CDKA is closely related to CDK1 in animals and to Cdc28 in *Saccharomyces cerevisiae* and Cdc2 in *Schizosaccharomyces pombe* and is expressed throughout the cycle. A second type of CDK, designated CDKB1 and CDKB2 are unique to plants [46,47] and active from S to M phase [48]. It is likely this unique plant feature controls whether the cell divides or enters reduplication without division; the presence of CDKB2 initiates cytokinesis, while in its absence endoreduplication takes place.

Phosphorylation of a plant homolog of the mammalian retinoblastoma protein initiates progression from G1 to S due to negative regulation of the E2F transcription factor family [49]. This is before DNA synthesis occurs. In S phase, the NE enlarges and the NPCs are replicated. These newly formed NPCs are inserted into the NE before NEBD. In yeast, three transmembrane proteins (Pom34, Pom152, and Ndc1) assemble first at the pore, followed by Nups59/53 and Nup170 [47,50]. While the assembly of the protein constituents of the pore has not been studied in plants, EM images of tobacco BY-2 cells revealing the development of the structure of NPCs show striking similarity to that in Xenopus [3]. Description of the process of NPC formation in plants is an important area for further exploration.

In M-phase, a unique plant structure, the preprophase band (PPB) made up of microtubules and F-actin, is formed as a ring predicting the plane of cell division [41]. This is in contrast to the actomyosin cortex surrounding the cytoplasm in dividing animal cells (see [51], for a review). In turn, its location has been preceded by a ring of RanGAP [41] at the plasma membrane. Microtubules, which in interphase had been surrounding the nuclear surface and radiating out to the cell cortex [52,53] based on the NE as a MTOC [54] and attached by the γ - tubulin complex [2], come together to form the PPB. Subsequently, the bipolar spindle is formed involving the formation of polar caps of MTs and associated with the γ-tubulin complex (see above). In *Arabidopsis*, the correct location of the spindle and phragmoplast microtubules during cell division involves GCP2, GCP4, and GCP-WD [55–58].

Nuclear envelope breakdown (NEBD) is key to open mitosis and involves the removal and relocation of the structures and components of the NE. NEBD in plants occurs earlier than in animals in late prophase [59]. The events resulting in NEBD are well described in animal systems (see [51] for a review). Briefly, rapid disintegration of the NPCs, with hyperphosphorylation of NUP98 results in permeabilization of the pore followed by the release of other pore components. Kinases influx during prophase, the NE dissociates from the lamins and chromatin resulting in the release of A-type lamins into the cytoplasm, while B-type lamins migrate to the endoplasmic reticulum. The LINC complex is directly involved in the dissociation of the NE from chromatin. SUN1 is phosphorylated on two sites by Cdk1 (serine 48 and 333) while Plk1 phosphorylates serine 138. SUN1 then loses interaction with partners binding to its N-terminal domain including the lamins, Emerin, and short nesprins. Thus, mitotic phosphorylation of SUN1 releases nucleoplasmic binding partners and permits disassembly of the lamina [60]. The interaction of the ONE with the cytoskeleton by the LINC complex has a second role, as the NE is disrupted by dynein-driven microtubule forces which act on the NE in the vicinity of centrosomes. Nuclear pore-based attachments to the cytoskeleton through RanBP2 and NUP 133 are also important in this process, with dynein driving tearing in pro-metaphase through LINC complex attachments (see [51]). The result of this is the migration of NE to join the pool of ER and exclusion of ER from mitotic chromatin. The ER is then enriched around the spindle poles, possibly forming a cap, either as sheets or tubules, the proportion varying within and between cell types [51], while NPCs migrate into stable subcomplexes and are not present as individual polypeptides [61,62]; some being degraded in a proteasome-dependent manner, perhaps to regulate pore numbers.

**Figure 1.**
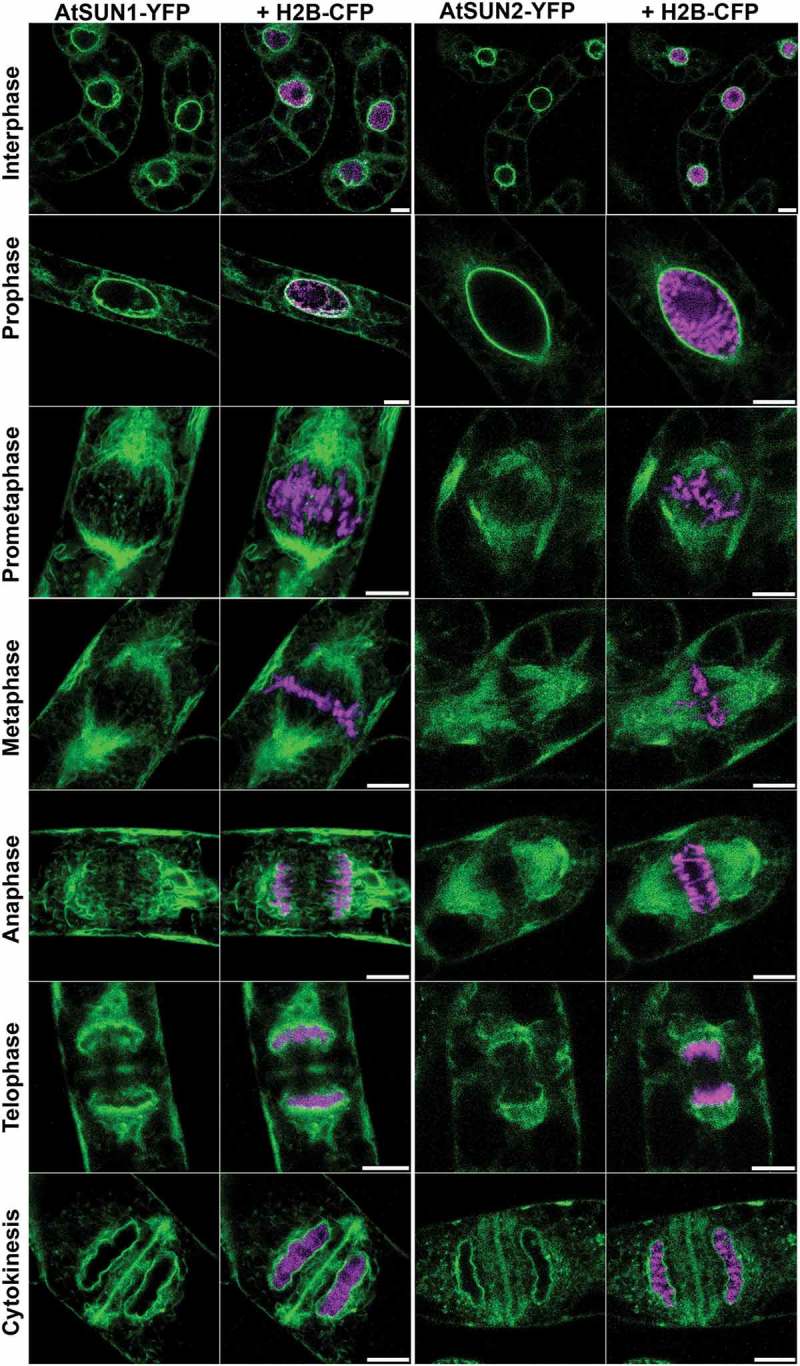
Subcellular localization of SUN domain proteins in synchronized, dividing BY-2 cells. Stable transformed BY-2 cells co-expressing either AtSUN1-YFP (green) and chromatin marker histone H2B-CFP (magenta) or AtSUN2-YFP (green) and H2B-CFP. The cells were synchronized using aphidicolin and living cells imaged by confocal microscopy. The two SUN proteins are present in the NE around chromatin in interphase and prophase. Upon NEBD, they distribute to mitotic ER and spindle membranes including tubules traversing the division zone. As the sister chromatids are separated, AtSUN1-YFP and AtSUN2-YFP accumulate in the reforming NE around chromatin first facing the spindle pole and finally proximal to the cell plate, to which they also localize. Scale bar = 10 μm. This figure was originally published in Graumann and Evans [42].

In higher plants, some similar events have been described (Figure 1). The pore basket component, NUA, relocates to the spindle in prometaphase [63]. Tobacco Rae1 co-localises with the preprophase band, mitotic spindle, and phragmoplast (newly forming cell plate) while deletion or downregulation of Rae1 results in a deformed spindle and mis-aligned chromatin [64]. Less is known about the behavior of the plant lamina components, though CRWN1 and CRWN2 disassemble after the onset of mitosis. Kimura et al. [65] showed that NMCP1 (CRWN1 family) migrates to the spindle and NMCP2 (CRWN4 family) to the cytoplasm in *Apium graveolens*. Association of the plant ONM with the cytoskeleton and a role in NEBD is suggested as NE tearing occurs where the nucleus is adjacent to the PPB and before the PPB breaks down [43,66-68].

Fluorescently labeled plant C-terminal SUN proteins AtSUN1 and AtSUN2 have been used by two laboratories to follow AtSUN1 and AtSUN2 in synchronized tobacco BY-2 cells [42,69]. From a uniform interphase NE distribution, AtSUN1-YFP and AtSUN2-YFP fluorescence decreases and then increases in intensity at the likely the sites of spindle pole formation and in the ER [42]. The NE can then be observed through the fluorescent labeling of AtSUN1 YFP. As NEBD begins in prometaphase, the NE is penetrated by spindle microtubules and then fragments [69]. By metaphase, NE is no longer visible and AtSUN1 locates to ER membranes around the spindle but not to obvious structures, such as spindle pole bodies (as described in [70]). Membranes containing AtSUN1-YFP appear as tubule-like structures with fluorescence around condensing chromatin; fluorescence was observed in anaphase surrounding segregated chromosomes [70]. Later, as the sister chromatids separate and migrate to the spindle poles, both AtSUN1-YFP and AtSUN2-YFP locate with decondensing chromatin facing the spindle poles, with some in tubule-like structures traversing the plane of division [42].The nucleoporin NUP88 (MOS7) is located at the NE in interphase in *Arabidopsis* and migrates to the spindle. It binds Rae1 and NUP98 (DRACULA). A* mos7* mutant shows failed spindle formation, defective cell-plate, and phragmoplast; as well as abortion of ovule and pollen [71,72].

## Reforming the nuclear envelope

In animal cells, the reformation of the NE begins with the surface of chromatin acting to recruit FG-rich nucleoporins (see [73] for a detailed review). The process involves a nucleosome template rich in DNA-histone. The Ran guanine exchange factor RCC1 causes an accumulation of RanGTP on the surface which likely results in attraction of cargo for the growing NE. In a reverse of the processes of nuclear envelope breakdown, histone is dephosphorylated by phosphatases and BAF is dephosphorylated allowing reassociation with LEM domain proteins. ER membrane containing NE proteins is therefore attracted to the surface of chromatin by the NE proteins within it, and the new daughter NE begins to form. During anaphase, as the NE begins to form around chromatin, NPCs begin to form with NUP133 and NUP153 first recruiting additional NPC components. Pre-assembled NPCs may also join the growing NE through the recruitment of membrane sheets to the growing NE [74].

Growth of the new NE is spatially regulated. Chromatin forms a disc-like structure perpendicular to the plane of division and can be divided into a peripheral ‘non core’, surrounding an inner core facing the division plane and an outer core facing away from it [73]. LBR, B-type lamins and SUN1 locate to the non-core outer region, where rapid NPC assembly also occurs, while LEM domain proteins, SUN2, and A-type lamins locate to the core regions. The envelope is then closed by membrane remodeling proteins which include a microtubule severing factor, spastin. Once the membrane is closed, additional NPCs form in the core region [75]

While evidence in plants is limited, two studies have shown that plant NE re-formation is spatially organized. The C-ter SUNs, AtSUN1, and AtSUN2 are first located to the surface of chromatin facing the spindle pole (i.e the outer core), then at the peripheral ‘non core’ and finally facing the cell plate (the division plane in plants) and therefore the inner core [42,69]. This organization was also observed for CRWN members NMCP1 and NMCP2 in *Apium graveolens* (celery). NMCP1 was first located on the chromatin surface facing the spindle (inner core) in late anaphase and then completely surrounded the chromatin at telophase [26]. Protein mobility assays showed that AtSUN2 remained strongly anchored to membranes throughout interphase and mitosis [42].

In contrast to animal cells, the dividing cells form a new cell wall to complete their separation. The presence of the wall prevents the swelling that occurs in animal cell division and the plane of the division has to be coordinated as the wall makes later correction impossible. As a result, large amounts of the membrane are involved not only in creating the two plasma membranes, but also in vesicle traffic to provide materials for the new wall. The initial structure, termed the cell plate, forms by centripetal expansion, growing outwards to meet the plasma membrane at the point initially predicted by the location of RanGAP and later by the pre-prophase band of microtubules. The cell plate and phragmoplast – the developing wall and membranes associated with it – contains AtSUN1 and AtSUN2 as well as the nuclear pore component Rae1, Ran, RanGAP, WITs, and WIPs [13,76,77]. The function (if any) of these is unknown; it may be simply a result of the very large activity of membrane growth and recovery taking place as the cell wall forms. However, a SUN2-YFP construct has a significant immobile fraction at the cell plate, suggesting that binding interactions are taking place [42] and mutants deficient for NUP88 (MOS7), a nucleoporin which binds Rae1 and NUP98, show disruption of the cell-plate and phragmoplast [71,72].

## Nuclear envelope proteins in meiosis

Interaction of chromosomes with the NE is essential during meiosis, the specialized cell division required for sexual reproduction. Meiosis involves a single S phase followed by two rounds of cell division that results in a halving of the chromosome number. The first meiotic stage, prophase I, is a relatively long period during which each chromosome must find its homologous partner within the nucleus, a process that involves large-scale chromosome movements, especially in organisms with large and complex genomes. These movements are driven by cytoplasmic forces, transmitted to chromosome ends by NE-associated proteins.

In interphase cells from big plant genomes (with C values above 4,000 Mbp, e.g. wheat, rye, barley, and oats) telomeres and centromeres cluster at opposite sides of the nucleus, facing previous division plane between the daughter cells. This is known as the Rabl organization (Rabl, 1885). This chromosome organization, however, is not present in plant genomes under 1,000 Mbp (e.g. sorghum, rice, Arabidopsis). In the specific case of Arabidopsis, there is a persistent telomere clustering at the nucleolus and no clustering of centromeres [78,79]. At the onset of meiosis, these configurations are lost, and during meiotic prophase (until pachynema) the telomeres are tightly clustered in the bouquet, in which chromosome ends are attached to the nuclear periphery [80–83]. Rabl organization involves the centromeres and the telomeres, whereas the bouquet seems to rely solely on the telomeres [84]. However, the size of the genome is not be the only factor determining the presence of Rabl conformation. Other plant species, such as maize (2,300 Mbp), despite presenting a large genome, are not known to exhibit Rabl organization at all [85].

**Figure 2.**
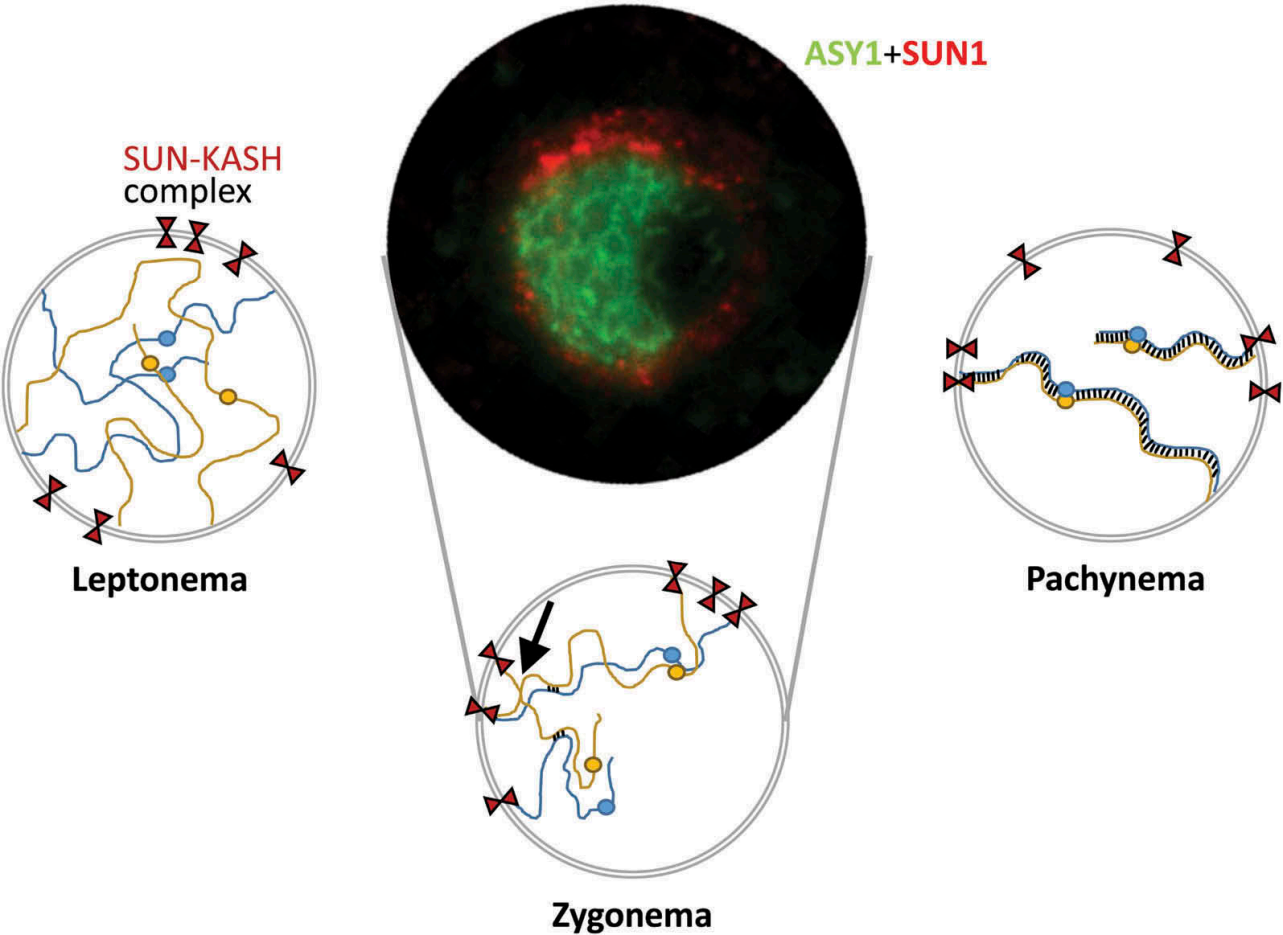
Chromosome dynamics during early prophase I. At leptonema, telomeres are attached to the nuclear periphery while homologous chromosomes align (pairing). At zygonema, the synaptonemal complex (SC) is assembled (synapsis). At this stage, the telomeres cluster in a limited region and form the bouquet. This configuration persists until pachynema when synapsis is complete. LINC complexes formed by SUN and KASH proteins are responsible for the attachment of telomeres to the NE and contribute to chromosome movements that facilitate homology searching and interlock resolution (arrow). In the middle of the figure, there is a zygotene meiocyte in which ASY1 (an axial element-associated protein, green) and SUN1 (red) are detected. The nucleolus appears to the right of the cell.

Pairing and synapsis are fundamental processes unique to meiotic chromosome dynamics. Pairing is mediated by recombinational interactions taking place in the context of chromosome structural axes and requires homology, whereas synapsis corresponds to installation of the synaptonemal complex (SC) between the chromosome axes, all along their lengths, and does not require homology [86]. The telomere bouquet precedes both pairing and synapsis and may facilitate these processes by positioning the subtelomeric regions of homologous chromosomes in close proximity and coalignment, through apparently both homology-dependent and homology-independent interactions [81,83]. In this context, SUN proteins have provided functional insight into telomere NE attachment during early meiotic prophase I. In mammals, SUN domain proteins interact with KASH5, a protein essential for bouquet formation, attached in turn to the microtubule dynein-dynactin complex in the cytoplasm [87,88]. Plant chromosome attachment to the NE also involves association with cytoskeletal elements tubulin and actin [89]. In common with animals, plant C-ter SUNs are involved in anchoring [7,8]. Varas et al. [8] were able to show that AtSUN1 and AtSUN2 localize to the NE in pollen mother cells (PMCs) in prophase I, in a pattern resembling telomere location (Figure 2) [90]. AtSUN2 was connected to the NE by a thread that resembled meiotic structures in yeast [90]. In an *Atsun1 Atsun2* double mutant the normally polarized location of telomeres to the NE in leptonema does not take place, prophase I is delayed, with incomplete synapsis and unresolved interlocks, there are univalents at metaphase I and missegregations at anaphase I that lead to the formation of aneuploid gametes [8]. Further evidence for a role for SUNs in telomere attachment in plants comes from maize, where ZmSUN2 forms a belt surrounding the meiotic cytoplasm that becomes a half-belt associated with the zygotene bouquet telomere structure [7] and meiotic mutants exhibit disruption of this belt. This strongly suggests that plant meiosis involves SUN domain telomere anchorage to the NE. A role for mid SUNs is also suggested in plants. In maize, ZmSUN3 has been proposed to play a role in meiotic divisions due to high levels of expression in pollen [91]. AtSUN5 is also expressed in pollen, although its meiotic function has not yet been demonstrated [6].

The LINC complex function in the attachment and repositioning of meiotic telomeres within the NE is widely conserved [92]. However, the possible involvement of other NE-associated proteins in coordinating the telomere-led chromosomal movements required to bring together the right chromosome pairs and to avoid wrong partners during prophase I remains obscure. Significant signs of progress in this issue are required to identify meiosis-specific factors and/or modifications involved in chromosome dynamics. For example, in *C. elegans* meiosis-specific phosphorylation of SUN-1 is essential for the efficient movement of meiotic telomeres [93,94], and the subsequent dephosphorylation is needed for further meiotic progression after pachytene [95]. In plants, the molecular mechanisms involved in the regulation of meiotic LINC complex function may be different. Furthermore, how telomeres are connected to the LINC complexes during plant meiosis is entirely unclear, because meiosis-specific adaptor proteins or factors that interact with SUN proteins in a meiosis-dependent manner have not been further identified [96]. In addition, many of the essential proteins involved in telomere organization do not have orthologous genes in the plant kingdom. On the other hand, it is still unknown whether CRWN proteins play a role in the association between telomeres and SUN proteins. Therefore, especially in plant meiosis, many questions regarding meiotic telomere attachment and movement still remain to be answered.

## Conclusion

Mitosis and meiosis in plants and animals achieve the same end results but show significant differences in mechanism and occur with different constraints and using different proteomes. The presence of the cell wall and the need to grow a new dividing wall to separate daughter cells require additional features. The LINC complex appears to have wider-ranging functions, especially in the localization of the RanGAP and the maintenance of the RanGTP concentrations needed to signal the location of chromatin and drive aspects of NE assembly.

It is evident that considerable further exploration of the plant system is needed to fully understand the processes of cell division, including the cell cycle control of the division, the breakdown and reformation of the NE, and the complex chromosome movements during meiosis. However, the identification of key component proteins, development and analysis of mutants and the use of live cell imaging have permitted significant progress and have high promise for future studies. What is clear from these studies is that although the composition of the NE across kingdoms is surprisingly diverse, the role of LINC complexes in plant cell division appears very similar to that observed in animals.
